# CLIMATE CHANGE AND EMERGENCY PREPAREDNESS FOR HURRICANES AMONG NURSING HOMES IN TEXAS

**DOI:** 10.1093/geroni/igad104.3728

**Published:** 2023-12-21

**Authors:** Brian Downer, Alexandra Holland, Yejin Kang, Briana Lacy, Huiwen Xu

**Affiliations:** University of Texas Medical Branch, Galveston, Texas, United States; UT Southwestern Medical Center, Dallas, Texas, United States; University of Texas Medical Branch, Galveston, Texas, United States; University of Texas Medical Branch, Galveston, Texas, United States; University of Texas Medical Branch, Galveston, Texas, United States

## Abstract

Hurricanes are a major threat to nursing homes along the Texas Gulf Coast, and climate change will increase inland nursing homes’ risk of catastrophic winds and flooding. The Centers for Medicare & Medicaid Services (CMS) uses twenty-five regulations to assess nursing homes’ emergency preparedness. This study compares the prevalence of any deficiencies in emergency preparedness regulations between Texas nursing homes in coastal and non-coastal counties. We also investigate the risk of future flood and wind events for nursing homes in non-coastal counties with any emergency preparedness deficiencies. Information for emergency preparedness deficiencies came from CMS inspection surveys. We used data from the First Street Foundation for the percentage of properties in a census tract with severe-extreme risk of flooding and damaging winds in 30 years. We used quartiles to define our highest risk categories as the census tracts with >7% of properties with severe-extreme risk for flooding and >50% with severe-extreme risk for damaging winds. The final sample included 1,153 nursing homes. The percentage of nursing homes with any deficiencies was significantly higher in non-coastal (13.9%) than coastal (8.7%) counties. This difference remained statistically significant after adjusting for ownership type, overall rating, and number of beds (OR=0.57, 95% CI=0.33-0.94). Among the 131 non-coastal nursing homes with any emergency preparedness deficiencies, 21.4% and 12.2% were in the highest risk categories for future flood and wind damage. Texas nursing homes, especially those in non-coastal counties, must be aware of their increasing hurricane risk and prepare to keep residents safe.

